# Examining the effect of stress on the flexible updating of avoidance responses

**DOI:** 10.1111/ejn.15155

**Published:** 2021-03-15

**Authors:** Anke Lemmens, Conny W. E. M. Quaedflieg, Pauline Dibbets, Marleen Rijkeboer, Tom Smeets

**Affiliations:** ^1^ Faculty of Psychology and Neuroscience Department of Clinical Psychological Science Maastricht University Maastricht The Netherlands; ^2^ Faculty of Psychology and Neuroscience Department of Neuropsychology & Psychopharmacology Maastricht University Maastricht The Netherlands; ^3^ CoRPS ‐ Center of Research on Psychological and Somatic disorders Department of Medical and Clinical Psychology Tilburg School of Social and Behavioral Sciences Tilburg University Maastricht The Netherlands

**Keywords:** acute stress, avoidance behavior, MAST, noradrenergic activity, reversal learning

## Abstract

Acute stress has been found to impair the flexible updating of stimulus − outcome associations. However, there is a lack of studies investigating the effect of acute stress on the flexible updating of stimulus–response associations, like active avoidance responses. The current study used an avoidance reversal learning paradigm to address this question. Sixty‐one participants learned that a red dot was associated with an aversive sound, whereas a green dot was not (Pavlovian Acquisition phase). Next, they were trained to avoid the aversive stimulus by selectively pressing a button in response to the red, but not the green, dot (Avoidance Acquisition phase). Subsequently, participants either underwent a stress induction task or a no‐stress control task. The flexible updating of expectancies of the US and avoidance responses were assessed after reversal of the original contingencies (Reversal Test). Acute stress did not impair the flexible updating of avoidance responses during the Reversal Test. In contrast, results showed that in the stress group the expectancies of the aversive sound were more in accordance with the reversed contingencies compared to the ratings of control participants. Additionally, cortisol responders avoided less often in comparison to cortisol non‐responders. Increased noradrenergic activity in stressed participants was related to impairments in the flexible updating of avoidance responses after contingency reversal, while this association was absent in the control participants. In conclusion, our results suggest that the autonomic response might account for shifting the balance toward inflexible updating of stimulus–outcome awareness while stress does not impair flexible updating of avoidance responses.

## Graphical abstract

Acute stress did not lead to more habitual avoidance responding. On the contrary, better stimulus–response awareness in a Reversal Test when under stress was observed. Furthermore, findings suggested that impairments in the flexible updating of avoidance responses are related to increased noradrenergic activity in stressed participants.

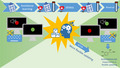

**Abbreviations**
CS: Conditioned Stimulus
DBP: Diastolic Blood Pressure
I‐PANAS‐SF: International Positive and Negative Affect Schedule Short‐Form
MAST: Maastricht Acute Stress Test
sAA: Salivary alpha‐amylase
SBP: Systolic Blood Pressure
SCR: Skin Conductance Responses
STAI: State‐Trait Anxiety Inventory
US: Unconditioned Stimulus

## 1 INTRODUCTION

Instrumental behavior, in which people learn that specific behaviors lead to specific desired outcomes, is controlled by a goal‐directed and a habitual regulatory system (De Wit & Dickinson, 2009; Dickinson, 1985; LeDoux & Daw, 2018). The goal‐directed system is driven by action–outcome associations. That is, our actions are based on the expected rewarding effects of the behavior, which can either be obtaining a positive outcome in case of appetitive conditioning or the omission of a negative outcome in aversive conditioning. An advantage of the goal‐directed system is that action selection can be optimized when the value of the outcome changes. Once the action–outcome relationships are established and the behavior is repeated, the habitual system might take over to guide action selection (Wood & Rünger, 2016). Habitual responses are not mediated by the anticipation of a goal, but by stimulus–response associations. That is, environmental stimuli automatically elicit the behavior when the association between the context and the response is strengthened through the repeated experience of a reward following the response (De Wit & Dickinson, 2009). Such associative learning can be highly adaptive as it enables us to react quickly and without using effortful cognitive resources. However, the inability to flexibly update stimulus − response associations that promote adaptive behavior can lead to maladaptive habitual behavior, such as persistent avoidance of stimuli that once were threatening, but now actually signal safety. In fact, inflexible responses to threat have been linked to many (neuro)psychiatric disorders such as obsessive‐compulsive disorder (Gillan et al., 2011, 2014, 2016; 2015; Voon et al., 2015), Gilles de la Tourette syndrome (Delorme et al., 2016), and substance dependence (Ersche et al., 2016; Gillan et al., 2016; Sjoerds et al., 2013; Voon et al., 2015).

Stress is known to be a risk factor for psychological disorders that are marked by inflexible responses to threat, and has been shown to affect habitual responding. The effect of stress on habitual responding has been studied extensively in appetitive instrumental learning paradigms (e.g., Quaedflieg et al., 2019; Schwabe & Wolf, 2009, 2010; Smeets et al., 2019), showing that acute stress induces a shift toward more habitual responding that is likely mediated by the stress hormones glucocorticoids and noradrenaline (Schwabe et al., 2010; Smeets et al., 2019; Wirz et al., 2018; Wood & Rünger, 2016). However, studies investigating the effect of stress on the flexible updating of avoidance responses, are sparse (Patterson et al., 2019; Raio et al., 2017). Using an aversive reversal learning paradigm, it was shown that acute stress induces a shift to habitual behavior control (Raio et al., 2017). In Raio et al.’s (2017) study, participants learned that one stimulus (conditioned stimulus; CS+) signaled an electric shock, whereas another stimulus (CS‐) signaled safety. One day later, participants received either an acute stress induction task or a control task and participants performed the task that they had learned the previous day, but unbeknownst to them the contingencies were now reversed. Acute stress resulted in reduced skin conductance responses (SCRs) to the new CS + during reversal learning (Raio et al., 2017). Interestingly, higher levels of noradrenergic activity (i.e., alpha‐amylase) were related to deficits in flexibly updating threat‐related stimulus–outcome associations (Raio et al., 2017). Furthermore, Patterson et al. (2019) conducted a study in which participants learned to make habitual avoidance responses to two warning stimuli that predicted aversive noise played to left and right earphones. In an outcome devaluation phase, participants were instructed to remove one of the two earphones. It was demonstrated that greater early‐life stress predicted greater odds of performing an avoidance habit after outcome devaluation (Patterson et al., 2019). Because avoidance behaviors can be repetitive by nature, it is important to study habitual avoidance and factors that increase these automatic behaviors.

Given that flexible responding is often required when under stress, the current study investigates whether acute stress leads to more habitual avoidance responses in healthy individuals. To our knowledge, only the study by Patterson and colleagues (2019) measured habitual avoidance responding to a devalued stimulus, but they asked participants to subjectively report on early‐life stress and did not manipulate stress experimentally. We developed an avoidance reversal learning paradigm that included overtraining of the avoidance response to elicit habitual avoidance. In the Pavlovian Acquisition phase, participants learned that one stimulus (red dot, CS+) was followed by an aversive sound, whereas another stimulus (green dot, CS‐) signaled safety. Pavlovian learning was followed by an Avoidance Acquisition phase in which participants were trained to selectively press a button in response to the red dot (CS+) in order to avoid the aversive stimulus, while not pressing to the green dot (CS‐). Following extensive training of the avoidance response, participants underwent either a stress induction task or a no‐stress control task. Habitual avoidance responding was assessed after reversal of the original contingencies (Reversal Test). Based on previous work showing that acute stress prompts habits (e.g., Quaedflieg et al., 2019; Schwabe & Wolf, 2009, 2010; Smeets et al., 2019), we expected that stress would lead to increased reliance on habitual avoidance, exhibited by impaired reversal learning in comparison to the no‐stress control group.

## 2 METHOD

### 2.1 Participants

A total of 64 individuals (aged 19–38, *M* = 21.75, *SD* = 2.81), 20 males and 44 females, participated in the current study. According to a power analysis (G*Power, repeated measures ANOVA, within‐between interaction) with η*_p_* ^2 = ^0.054 (Raio et al., 2017), power = 0.80, two groups (between‐subjects), and four blocks (within‐subjects), *N* = 68 participants had to be included in the study in order to make statistical inferences with sufficient power. Participants were all students enrolled in the second year of the Bachelor Psychology (Faculty of Psychology and Neuroscience; Maastricht University) and took part in a course on research skills. Exclusion criteria were self‐reports of (1) a psychological disorder diagnosis and/or receiving treatment for a psychological disorder at the time of or within three years before participation, (2) cardiovascular diseases, (3) pregnancy, (4) red‐green color‐blindness, and (5) insufficient hearing (i.e., not restored through hearing aids). Before the start of the experiment, participants signed a written informed consent and were pseudo‐randomly (i.e., equal male‐to‐female ratio in each group) allocated to the stress or no‐stress control group. After completion of the experiment, participants were compensated with 1.5 course credits. The study was approved by the Ethics Review Committee Psychology and Neuroscience at Maastricht University (ERCPN‐205_10_03_2019 and RP2027_2019_34).

## 3 MATERIALS

### 3.1 Habitual avoidance paradigm

**FIGURE 1:**
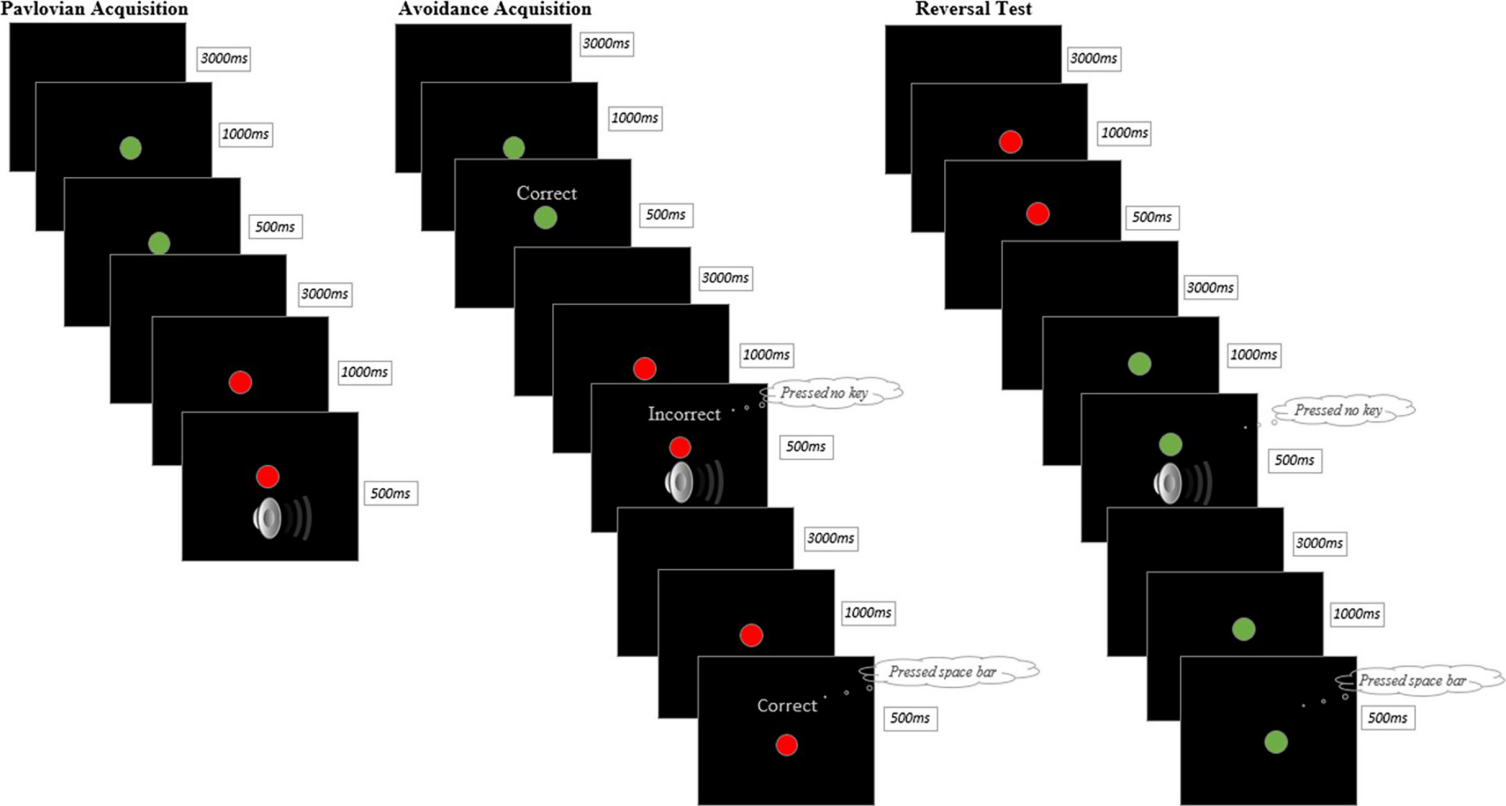
Overview of the habitual avoidance paradigm

The paradigm that was developed for the current study consisted of three phases: A Pavlovian Acquisition phase, an Avoidance Acquisition phase, and a Reversal Test. Two colored dots presented against a black background served as the CS during our experimental task: a red dot (CS+) and a green dot (CS‐). The unconditioned stimulus (US) was a 500‐ms loud female scream (100 dB). An overview of the task is presented in Figure 1.

During the habitual avoidance task, participants completed ***US‐expectancy ratings*** repeatedly namely (#1) before the Pavlovian Acquisition phase, (#2) in between block 1 and 2 of the Pavlovian Acquisition phase, (#3) after the Pavlovian Acquisition phase, (#4) after the Avoidance Acquisition phase, (#5) after the stress induction or control procedure but before the Reversal Test (see below), and (#6) after the Reversal Test. Using a slider, they indicated to what extent they expected that the red and green dot would be followed by the sound on a scale from “‐5 ‐ for sure no sound” to “+5 ‐ for sure sound”, with “0 – uncertain” as midpoint of the scale. Ratings were recoded to 0 – 100, similar to the majority of Pavlovian fear conditioning studies. From the Avoidance Acquisition phase onwards, the question was changed into “*If you would not press the space bar, would you expect that the red/green dot will be followed by the sound?*” in order to assess US expectancies irrespective of whether or not participants avoided the sound. Finally, as a manipulation check, participants were asked to rate whether they thought the space bar was effective in preventing the sound on a scale from “‐5 – never” to “+5 – always”, with “0 – uncertain” as midpoint of the scale (again recoded to 0–100). Participants completed the avoidance effectiveness ratings after the Avoidance Acquisition phase and after the Reversal Test.

### 3.2 Pavlovian acquisition phase

The Pavlovian Acquisition phase consisted of two blocks of five CS+ and five CS‐ trials each that were presented in random order. The only restriction was no more than two consecutive trials for the same stimulus. The CS+ was followed by the US in 80% of the trials, whereas the CS‐ was never followed by the scream. Participants were instructed to monitor the relations between the stimuli and their consequences. Each trial started with a black screen lasting for three seconds, followed by a 1.5 s colored dot. During the last 500 ms of CS presentation on CS+ trials, the US was presented.

### 3.3 Avoidance acquisition phase

In the Avoidance Acquisition phase, the US followed in 100% of the CS+ trials, unless participants pressed the space bar. Participants were instructed that the color–sound relationships remained the same and that they could now avoid the sound by pressing the space bar. They were told to avoid as much as possible, but only on trials for which they expected the aversive sound. The US could be avoided by pressing the space bar within 1000 ms after the CS was presented. In the first block (10 CS+ and 10 CS‐ trials, randomly presented), participants received immediate feedback on their performance. If they pressed the space bar within 1000 ms after the red dot was presented or refrained from pressing the space bar after the green dot was presented, the feedback “Correct” (and additionally the reaction time in case of a red dot) appeared on the screen during the final 500 ms of CS presentation. Whenever participants pressed the space bar in response to the green dot or refrained from pressing the space bar in response to the red dot, the feedback “Incorrect” or “Try to respond faster”, respectively, appeared on the screen during the final 500 ms of CS presentation. The phase continued with a block consisting of 20 CS+ trials without immediate feedback in order to decrease trial duration and install an avoidance habit. After the second block, four more blocks consisting of 20 trials without immediate feedback (10 CS+ and 10 CS‐ trials, randomly presented) followed in order to over‐train the avoidance response, thereby strengthening the habitual nature of the avoidance response. Again, the order was restricted to two consecutive trials for the same stimulus. In between block 2 and 3 and between block 4 and 5, the instruction that participants should avoid as much as possible but only on trials for which they expected the aversive sound, was repeated.

### 3.4 Reversal test

To measure flexible updating, stimulus–response contingencies of the CS+ and CS‐ were reversed. The green dot was now followed by the scream and the red dot was not. Again, unless participants pressed the space bar the US followed CS presentation in 100% of the new CS+ trials. Participants were not informed about this reversal. Similar to the Avoidance Acquisition phase, participants were instructed at the start of the Reversal Test that they could avoid the sound by pressing the space bar and to avoid as much as possible, but only on trials for which they expected the aversive sound. The Reversal Test consisted of ten blocks of four trials (2 CS+ and 2 CS‐ trials). After block 2, 5, and 7 participants could take a short break before continuing to the next blocks. The avoidance instruction was not repeated during the Reversal Test.

## 4 PHYSIOLOGICAL AND NEUROENDOCRINE STRESS RESPONSES

### 4.1 Stress manipulation

The Maastricht Acute Stress Test (MAST; Smeets et al., 2012; Quaedflieg et al., 2017; see also Shilton et al., 2017) was used to induce acute stress. The task started with a 5‐min preparation phase in which the task was explained to the participants. During the 10‐min acute stress phase, participants were repeatedly exposed to cold pressor stress and performing mental arithmetic challenges. More specifically, they had to immerse their non‐dominant hand into a plastic box with ice‐cold water (4°C) during five trials of different durations (60 – 90 s). In between the hand immersion trials, participants had to count backwards as fast and accurately as possible in steps of 17 starting at four different random numbers, for example, 2043 (45 – 90 s). To further increase stress levels, participants were told that they were videotaped during the task and saw themselves on a monitor. Also, they received negative feedback (e.g., “count faster” or “incorrect, start over again”) when they engaged in the calculations. To increase uncontrollability, participants were told that the order and duration of the hand immersion and mental arithmetic trials would be randomly chosen by the computer.

The no‐stress control task followed a similar procedure than the MAST, except that all stressful elements were removed. More specifically, participants had to immerse their hand into lukewarm water (35°C), instead of difficult mental arithmetic challenges they had to count aloud from 1 to 25, and they were not videotaped and did not see themselves. Even though the experimenter was present in the laboratory, he/she provided no feedback on the performance of the participants.

After the MAST or no‐stress control task, participants had to rate their subjective stress levels by indicating how painful, unpleasant and stressful the just performed task was for them on a VAS‐scale from “0 – not stressful at all” to “100 ‐ very stressful”.

### 4.2 Blood pressure

Systolic (SBP) and diastolic (DBP) blood pressure were measured using a fully automated upper‐arm oscillometric blood pressure monitoring device (Omron 705IT; HEM‐759‐E; Omron Healthcare Europe BV, Hoofddorp, the Netherlands). SBP and DBP were assessed 5 min before the start of the MAST or no‐stress control task, during the second hand immersion trial of the MAST and immediately after the end of the MAST or no‐stress control task.

### 4.3 Alpha‐amylase and cortisol

Salivary measurements with synthetic Salivettes (Sarstedt®, Etten‐Leur, the Netherlands) were obtained assessing salivary alpha‐amylase (sAA) as a measure of the fast, noradrenergic stress response (Nater & Rohleder, 2009; Strahler et al., 2017) and cortisol as a measure of the slower, HPA axis response. Participants provided saliva samples prior to the MAST or no‐stress control task, and two times afterwards (t_+10_, t_+20_ min with reference to the end of the stressor). Samples were stored at − 20°C until alpha‐amylase and cortisol levels were determined by a commercially available luminescence immune assay kit (IBL, Hamburg, Germany). Mean intra‐ and inter‐assay coefficients of variation were below 10% for both analyses.

## 5 QUESTIONNAIRES

### 5.1 International Positive and Negative Affect Schedule Short‐Form (I‐PANAS‐SF)

We used the International Positive and Negative Affect Schedule Short‐Form (Thompson, 2007) to measure subjective stress. The I‐PANAS‐SF consists of two 5‐item scales to measure both positive (e.g., attentive) and negative affect (e.g., hostile). Items are derived from the original 20‐item PANAS (Watson et al., 1988). Participants were asked to indicate to what extent they felt the different feelings and emotions at the present moment on a 5‐point Likert scale ranging from “1 ‐ not at all” to “5 – extremely”. Validation studies of the I‐PANAS‐SF have demonstrated that the scale was psychometrically acceptable, based on examinations of the cross‐sample stability, internal reliability, temporal stability, cross‐ cultural factorial invariance, and convergent and criterion‐related validity (Thompson, 2007).

### 5.2 State‐Trait Anxiety Inventory (STAI)

The STAI (Spielberger et al., 1970) is a 40‐item self‐report measure of state and trait anxiety. Both the STAI‐T(rait) and STAI‐S(tate) subscales consist of 20 items. In the current experiment, we were interested in subjective state anxiety (STAI‐S). Participants had to rate to what extent the items reflected how they felt at that moment on a 4‐point Likert scale ranging from “1 ‐ not at all” to “4 ‐ very much so”. The STAI has demonstrated satisfactory psychometric properties (Barnes et al., 2002).

## 6 Procedure

**FIGURE 2:**
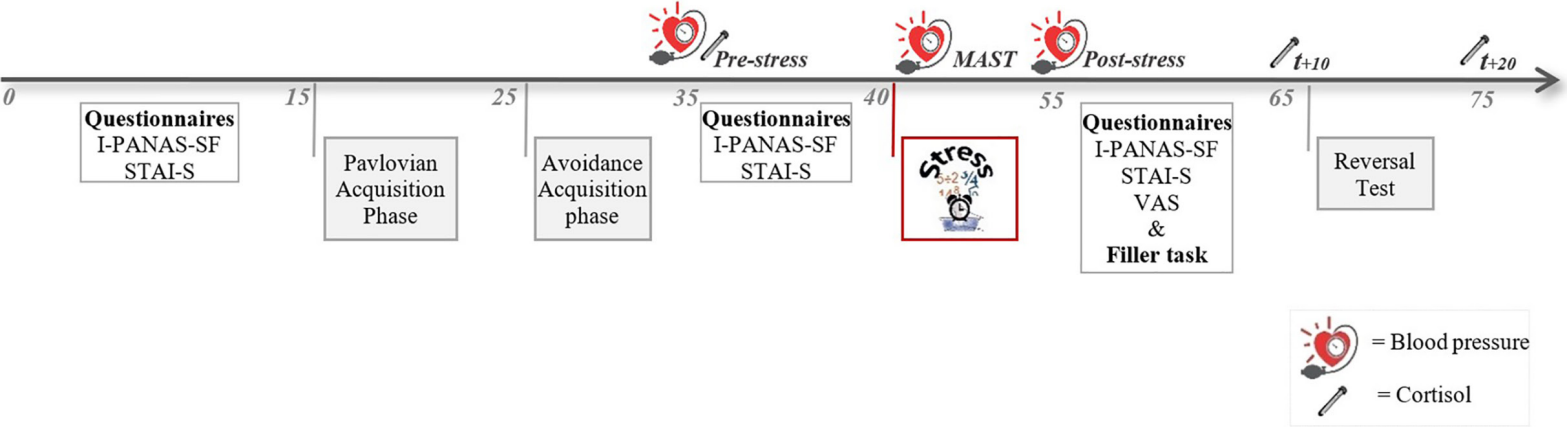
Overview of the experimental procedure. Time in minutes

Figure 2 displays an overview of the experimental procedure. The day before the experiment took place, participants received instructions via email. They were kindly invited to eat breakfast, but to refrain from eating, smoking, exercising, and drinking anything except from water two hours before the start of the experiment. They were also kindly invited to take the elevator instead of the stairs (i.e., to minimize arousal effects) at the day of the experiment. Testing days ran between 11:00 a.m. and 8:00 p.m. in order to minimize morning fluctuations in cortisol levels. Upon arrival in the laboratory, adherence to the instructions was checked by the experimenter and participants were presented with an information letter and provided informed consent. Subsequently, the experimental procedure started (as depicted in Figure 2). Blood pressure and cortisol measurements were taken simultaneously. To ensure that 10 min would pass after the end of the stress induction, a filler task (i.e., Digit Span Task; Wechsler, 1981) was added between the MAST and the post‐stress cortisol measurement (t_+10_). Both the Forward and Backward version of the Digit Span Task were administered for 10 min after the stress manipulation, where after the filler task was terminated. At the end of the experiment, participants were debriefed and compensated for their participation.

## 7 Data analysis

Data analyses were performed using SPSS Statistics for Mac, version 25 (SPSS Inc., Chicago, Ill., USA). The data were checked for normality and outliers. *P*‐values were corrected using Greenhouse‐Geisser estimates of sphericity when required. All reported *p*‐values are two‐tailed, unless stated otherwise. The standard rejection criterion was set at *p* <.05 throughout. Partial Eta Squared (η*_p_* ^2^) values were reported as a measure of effect size for statistically significant results. Significant (interaction) effects were followed up with pairwise comparisons or post hoc analyses. In case of multiple comparisons, Bonferroni corrections were applied.

Contingency awareness was checked as avoidance and reversal learning are inconsequential when no contingency learning has occurred. More specifically, participants were excluded from analyses if they rated the US expectancy of the green dot higher than that of the red dot after the Pavlovian Acquisition Phase. Regarding the Reversal Test, participants were excluded from the analyses if they adopted a better‐safe‐than‐sorry strategy. That is, if participants pressed the space bar during 100% of the Reversal Test trials with the red dot (i.e., new CS‐). After excluding participants, a randomization check was performed, comparing demographic variables, questionnaire scores, and the baseline US‐expectancy ratings using ANOVAs and χ^2^ ‐tests.

We investigated the effectiveness of the stress induction procedure by examining subjective stress ratings (painfulness, pleasantness, stressfulness) after the MAST using a GLM Multivariate ANOVA. The effect of the MAST on negative affect scores (I‐PANAS‐SF‐NA), state anxiety scores (STAI‐S), blood pressure, salivary alpha‐amylase, and cortisol levels were assessed using GLM repeated measures ANOVAs. Cortisol and sAA data were log‐transformed due to typical skewness of the data. As it is known that not all individuals respond with increases in glucocorticoid responses to the MAST (Quaedflieg et al., 2017; Smeets et al., 2019), we calculated a cortisol responder rate, representing participants with a cortisol increase equal to or larger than 1.5 nmol/l relative to pre‐stress (Miller et al., 2013). Fifty‐two percent of the participants in the stress condition (16 out of 31) were classified as cortisol responders. A GLM repeated measures ANOVA with ResponderType (control versus. responder versus. non‐responder) as between‐subjects factor was used to demonstrate significant ResponderType differences in cortisol responses.

US‐expectancy ratings were analyzed for each phase separately using GLM repeated measures ANOVAs. Not only the effect of Group was investigated, but we also performed similar GLM repeated measures analyses with ResponderType as between‐subjects variable as we were interested in the distinct effect of high versus low glucocorticoid stress responses. Percentages of avoidance responses during the final block of the Avoidance Acquisition phase were calculated in order to check whether the paradigm was successful in eliciting differential avoidance responses to the CS+ and CS‐. Again, the analysis was repeated with ResponderType as between‐subjects variable. In line with Raio and colleagues (2017), only the first 12 CS‐ (red dot) and CS+ (green dot) trials of the Reversal Test were analyzed in four blocks of three trials to detect habitual responding and reversal learning. We started counting avoidance responses after participants were exposed to the first green trial, because on this trial they could for the first time learn that contingencies had been reversed. Next, we calculated percentages of avoidance responses to the new CS‐ and CS+ in the separate blocks of three trials. A GLM repeated measures ANOVA was used to investigate habitual avoidance responding. To assess whether possible increased habitual avoidance responding could be accounted for by glucocorticoid responses in the stress condition, a GLM repeated measures ANOVA with ResponderType as between‐subjects variable was conducted.

Finally, as Raio and colleagues (2017) found a significant Group effect on the “reversal index” and significant correlations between alpha‐amylase levels and the “reversal index”, we conducted similar analyses. The reversal index in the study by Raio et al. (2017) reflected the difference in the magnitude of CRs between the reversal and acquisition phase (i.e., mean reversal CR minus mean acquisition CR). In the current study, we were interested in US expectancies and avoidance responses. Therefore, we calculated (a) a reversal index reflecting the difference in US‐expectancy ratings between the green dot after the Reversal Test and the red dot after the Avoidance Acquisition phase, and (b) a reversal index reflecting the difference in percentage of avoidance responses between the Reversal Test (12 CS+ trials) and the final block of the Avoidance Acquisition phase (10 CS+ trials). We performed one‐way ANOVAs of Group and ResponderType on the reversal indices and calculated Bivariate Pearson correlations per group between the physiological and neuroendocrine responses and the reversal indices. The Holm‐Bonferroni method was used to correct for multiple comparisons in the correlational analyses (Holm, 1979).

## 8 RESULTS

### 8.1 Included Sample

Three participants (1 male, 2 female; 1 in stress condition, 2 in control condition) were excluded from the analyses, one due to data storage failure (1 female in the control condition) and two because they adopted a better‐safe‐than‐sorry strategy (cf. supra; 1 male in the control and 1 female in the stress condition). All remaining participants were contingency aware. Hence, the total sample consisted of *N* = 61 (control: *n* = 30; stress: *n* = 31) participants. Groups did not differ in gender ratio (χ^2^ (*N* = 61) = 0.13, *p* =.72), age, baseline STAI‐S, and baseline I‐PANAS‐SF‐NA scores (*F*s < 0.05, *p*s > 0.82). There was also no difference between groups in baseline US expectancy of the red and green dot (*F*s < 2.08, *p*s > 0.15).

### 8.2 Stress manipulation

**FIGURE 3:**
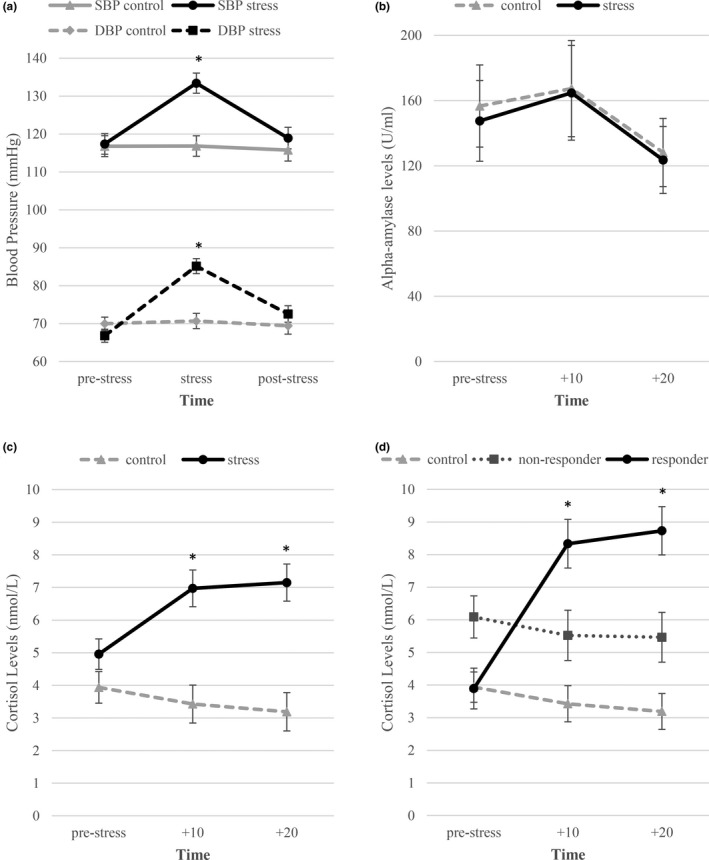
FIGUREThe physiological stress response. (a) Mean systolic and diastolic blood pressure levels (± SE) for the stress and control group. (b) Mean raw alpha‐amylase levels (± SE) for the stress and control group. (c) Mean raw cortisol levels (± SE) for the stress and control group. (d) Mean raw cortisol levels (± SE) for the cortisol responder groups. Significant group differences are marked, * *p* < 0.05

To verify the stress manipulations, we conducted mixed ANOVAs on the effect of Group (stress versus. no‐stress control) on subjective stress, negative affect, state anxiety, systolic and diastolic blood pressure, salivary alpha‐amylase, and cortisol levels. Table 1 provides an overview of the descriptive and main inferential statistics and Figure 3 graphically represents the data of the stress manipulation checks. Participants in the stress condition perceived the MAST as distressing, indicated by their higher ratings of subjective stress, negative affect, and state anxiety in comparison to the no‐stress control group (*F*s > 15.79, *p*s < 0.001).

**TABLE 1:** Inferential statistics and means (± SE) of subjective stress, negative affect, state anxiety, blood pressure levels, salivary alpha‐amylase, and cortisol levels per Group and ResponderType

				Stress (*n* = 31)									No‐stress control (*n* = 30)										Main effect Group									
Subjective Stress				Mean (± SE)									Mean (± SE)										*F, p*									
Painfulness				50.87 (5.96)									15.30 (5.32)										19.74, *p* < 0.001									
Unpleasantness				68.84 (5.64)									34.70 (6.50)										15.79, *p* < 0.001									
Stressfulness				51.19 (5.60)									17.37 (5.11)										19.83, *p* < 0.001									
																							**Interaction Group x Time**									
				**Pre‐stress** Mean (± SE)			**Post‐stress** Mean (± SE)						**Pre‐stress** Mean (± SE)				**Post‐stress** Mean (± SE)						**Pre‐stress** ***F, p***					**Post‐stress** ***F, p***				
Negative affect				7.32 (0.56)			9.03 (0.71)						7.27 (0.45)				5.97 (0.29)						0.006, *p* = 0.94 0.25, *p* = 0.62					15.83, *p* < 0.001				
State anxiety			37.10 (1.94)				44.55 (2.18)						38.47 (1.96)				33.03 (1.47)											18.91, *p* < 0.001				
				**Pre‐stress** Mean (± SE)		**Stress** Mean (± SE)				**Post‐** **stress** Mean (± SE)			**Pre‐stress** Mean (± SE)			**Stress** Mean (± SE)			**Post‐stress** Mean (± SE)				**Pre‐stress** ***F, p***		**stress** ***F, p***					**Post‐stress** ***F, p***		
**SBP**				117.39 (2.79)		133.42 (2.86)				118.94 (3.13)			116.80 (2.67)			116.83 (2.45)			115.77 (2.55)				0.02, *p* = 0.88		19.22, *p* < 0.001					0.61, *p* = 0.44		
**DBP**				66.81 (1.80)		85.16 (2.10)				72.55 (2.14)			69.97 (1.66)			70.70 (1.88)			69.43 (2.24)				1.66, *p* = 0.20		26.22, *p* < 0.001					1.02, *p* = 0.32		
				**Pre‐stress** Mean (± SE)		**t + 10** Mean (± SE)			**t + 20** Mean (± SE)				**Pre‐stress** Mean (± SE)		**t + 10** Mean (± SE)					**t + 20** Mean (± SE)			**Pre‐stress** ***F, p***				**t + 10** ***F, p***			**t + 20** ***F, p***		
**Ln sAA**				4.60 (0.18)		4.87 (0.14)			4.58 (0.14)				4.68 (0.17)		4.67 (0.18)					4.44 (0.17)			0.11, *p* = 0.74			0.83, *p* = 0.37				0.40, *p* = 0.53		
**Ln Cortisol**				1.41 (0.12)		1.80 (0.10)			1.82 (0.10)				1.29 (0.12)		1.16 (0.11)					1.10 (0.11)			0.47, *p* = 0.49			18.45, *p* < 0.001					24.07, *p* < 0.001	
		**Responder (*n* = 16)**										**Non‐responder (*n* = 15)**									**Control (*n* = 30)**						**Interaction Group x Time**					
	**Pre‐stress** Mean (± SE)				**t + 10** Mean (± SE)			**t + 20** Mean (± SE)			**Pre‐stress** Mean (± SE)			**t + 10** Mean (± SE)		**t + 20** Mean (± SE)		**Pre‐stress** Mean (± SE)				**t + 10** Mean (± SE)		**t + 20** Mean (± SE)			**Pre‐stress** ***F, p***		**t + 10** ***F, p***			**t + 20** ***F, p***
**Ln Cortisol**	1.20 (0.15)				1.98 (0.14)			2.04 (0.13)			1.63 (0.16)			1.61 (0.12)		1.59 (0.13)		1.29 (0.12)				1.15 (0.11)		1.10 (0.11)			2.03, *p* = 0.14		11.22, *p* < 0.001			15.39, *p* < 0.001

Note

Negative affect was measured using the I‐PANAS‐SF‐NA and state anxiety using the STAI‐S. Subjective stress ratings after the MAST were analyzed using a GLM Multivariate ANOVA with Group as between‐subjects variable. I‐PANAS‐SF‐NA and STAI‐S scores, systolic and diastolic blood pressure levels (SBP and DBP), log‐transformed salivary alpha‐amylase (sAA), and log‐transformed cortisol levels were analyzed using GLM repeated measures ANOVAs with Group as between‐subjects variable and Time as within‐subjects variable. The analysis of the log‐transformed cortisol levels was repeated with ResponderType as between‐subjects variable.

Blood pressure measures confirm physiological arousal induced by the MAST. Stress differentially affected both systolic and diastolic blood pressure depending on the timing (see Table 1). Follow‐up tests revealed no statistically significant differences between groups at baseline (all *p*s ≥ 0.20, see Table 1). During the stressor, the stress group showed statistically significantly higher blood pressure than the non‐stressed group (all *p*s ≤ 0.001, see Figure 3a). With respect to alpha‐amylase levels, groups did not differ per timing (see Figure 3b). The main effect of time revealed a quadratic trend (*p* =.01) indicating that sAA increased during the MAST and decreased thereafter.

Salivary cortisol levels confirm the acute stress induction. Stress differentially affected salivary cortisol levels as a function of timing (see Table 1). Follow‐up tests revealed no statistically significant differences in salivary cortisol between stress conditions at baseline (*p* =.49, see Table 1). After the stressor, the stress group showed statistically significantly higher salivary cortisol levels than the non‐stressed group at both time points (*p*s < 0.001, see Table 1 and Figure 3c). The analyses with responder type revealed the same results (see Table 1 and Figure 3d).

### 8.3 Habitual avoidance learning

To verify the fear and avoidance learning, we conducted mixed ANOVAs on the effect of Group (stress versus. no‐stress control) on expectancy ratings for both CS+ and CS‐. Table 2 provides an overview of the descriptive statistics.

**TABLE 2:** Means (± SE) of US‐expectancy ratings, percentage avoidance responses, and avoidance effectiveness ratings per Group and Responder Type during the Reversal Test phase

		Stress (*n* = 31)		No‐stress control (*n* = 30)		Responder (*n* = 16)			Non‐responder (*n* = 15)			
		CS+	CS‐	CS+	CS‐	CS+	CS‐		CS+		CS‐	
		Mean (± SE)	Mean (± SE)	Mean (± SE)	Mean (± SE)	Mean (± SE)	Mean (± SE)		Mean (± SE)		Mean (±SE)	
**US expectancy**												
Baseline	#1	58.39 (2.44)	41.68 (2.88)	54.57 (2.90)	47.27 (2.59)	59.25 (3.66)		40 (3.57)	57.47 (2.90)		43.47 (4.65)	
Pavlovian Acquisition	#2	87.06 (1.65)	7.97 (2.55)	82.50 (2.09)	9.30 (2.45)	85.50 (1.96)		4.19 (1.78)	88.73 (2.70)		12.00 (4.78)
	#3	89.77 (1.85)	5.77 (2.19)	85.67 (1.75)	11.37 (2.74)	91.50 (1.91)		1.75 (1.38)	87.93 (3.25)		10.07 (4.06)
Avoidance Acquisition	#4	77.06 (4.07)	10.68 (3.95)	79.93 (3.80)	7.40 (2.58)	80.25 (4.96)		4.06 (1.90)	73.67 (6.60)		17.73 (7.63)
**Avoidance responses**		92.47 (1.24)	10.75 (2.63)	91.94 (1.35)	12.22 (2.29)	91.15 (1.61)		6.25 (1.94)	93.89 (1.90)		15.56 (4.80)
**Avoidance effectiveness**		90.90 (3.07)		90.57 (3.89)		94.94 (3.40)				86.60 (5.10)		

Note

Expectancy *r*ating 4 reflects whether participants would expect the sound if they would not press the button.

For the Pavlovian Acquisition phase, US‐expectancy ratings confirmed differential fear learning. The main effect of stimulus (*F*(1,59) = 887.14, *p* <.001, η*_p_* ^2^ = 0.94) demonstrated that participants expected the sound after the red and not after the green dot. There were no differences between groups or responder types (*F*s < 3.01, *p*s > 0.07) during fear acquisition.

For the Avoidance Acquisition phase, avoidance responses and expectancy ratings confirmed successful differential learning. The percentages of avoidance responses revealed that participants pressed the space bar in 99.84% in response to the CS+ and in < 1% in response to the CS‐ during the final Avoidance Acquisition block. This was corroborated by the avoidance effectiveness ratings showing that participants learned that pressing the space bar would avoid the sound (*M* = 90.74). In addition, US‐expectancy ratings regarding what participants expected if they did not avoid (i.e., press the space bar), confirmed that participants still expected the sound after the red and not after the green dot (*F*(1,59) = 302.00, *p* <.001, η*_p_* ^2^ = 0.84). There were no differences between groups or responder types during the Avoidance Acquisition phase for responding and expectancy (*F*s < 2.00, *p*s > 0.14).

### 8.4 Habitual avoidance — reversal test

**FIGURE 4:**
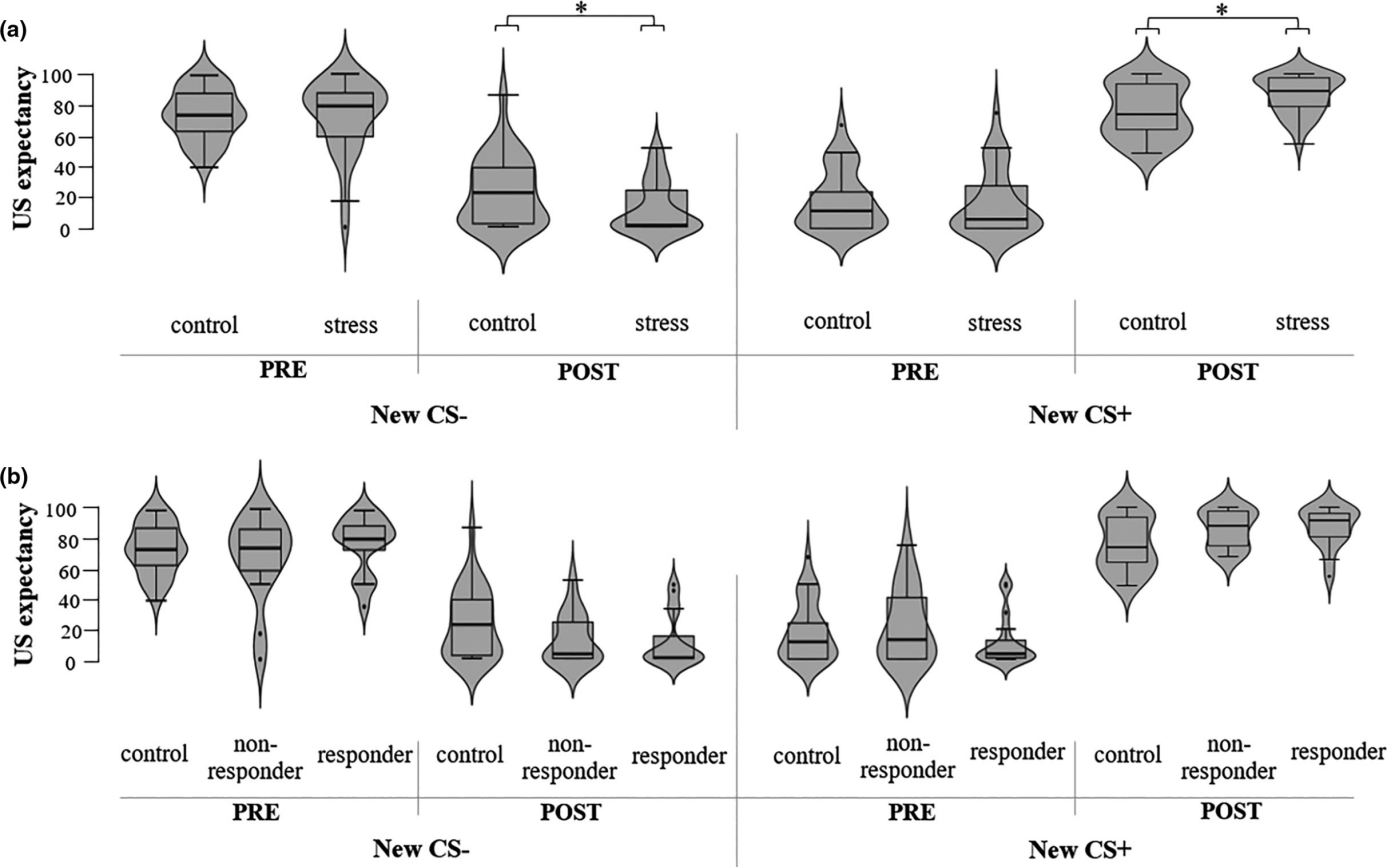
US‐expectancy ratings for the new CS‐ (red) and CS+ (green) (± SE) before and after the Reversal Test. (a) Comparison stress and control group, (b) Comparison controls, cortisol non‐responders and responders. Significant group differences are marked, * *p* < 0.05

To examine the effect of stress on habitual avoidance, we assessed US‐expectancy ratings and avoidance responding during the Reversal Test. For the expectancy ratings, the mixed ANOVA revealed a significant Group*Stimulus interaction (*F*(1,59) = 7.63, *p* =.008, η*_p_* ^2^ = 0.11). Follow‐up tests revealed that groups differed significantly in US‐expectancy ratings of both the red (new CS‐: *F*(1,59) = 4.92, *p* =.03) and green dot (new CS+: *F*(1,59) = 7.46, *p* =.01) after the Reversal Test. Expectancy ratings of participants in the stress group (CS‐: *M* = 11.90, *SE* = 3.04; CS+: *M* = 86.58, *SE* = 2.24) were closer to the actual contingencies (i.e., new CS‐: 0%, new CS+: 100%) in comparison to ratings of no‐stress control participants (CS‐: *M* = 23.30, SE = 4.11; CS+: *M* = 76.07, *SE* = 3.16; see Figure 4a). The analyses with responder type revealed also a significant ResponderType*Stimulus interaction (*F*(2,58) = 3.79, *p* =.028, η*_p_* ^2^ = 0.12). Follow‐up tests showed that there was a significant difference between the responder groups for the green dot (new CS+: *F*(2,58) = 3.69, *p* =.031), but not for the red dot (new CS‐: *F*(2,58) = 2.45, *p* =.95; see Figure 4b). However, pairwise comparisons revealed no significant differences in US‐expectancy ratings of the green dot between the responder groups when applying Bonferroni corrections (*p*s > 0.065).

**FIGURE 5:**
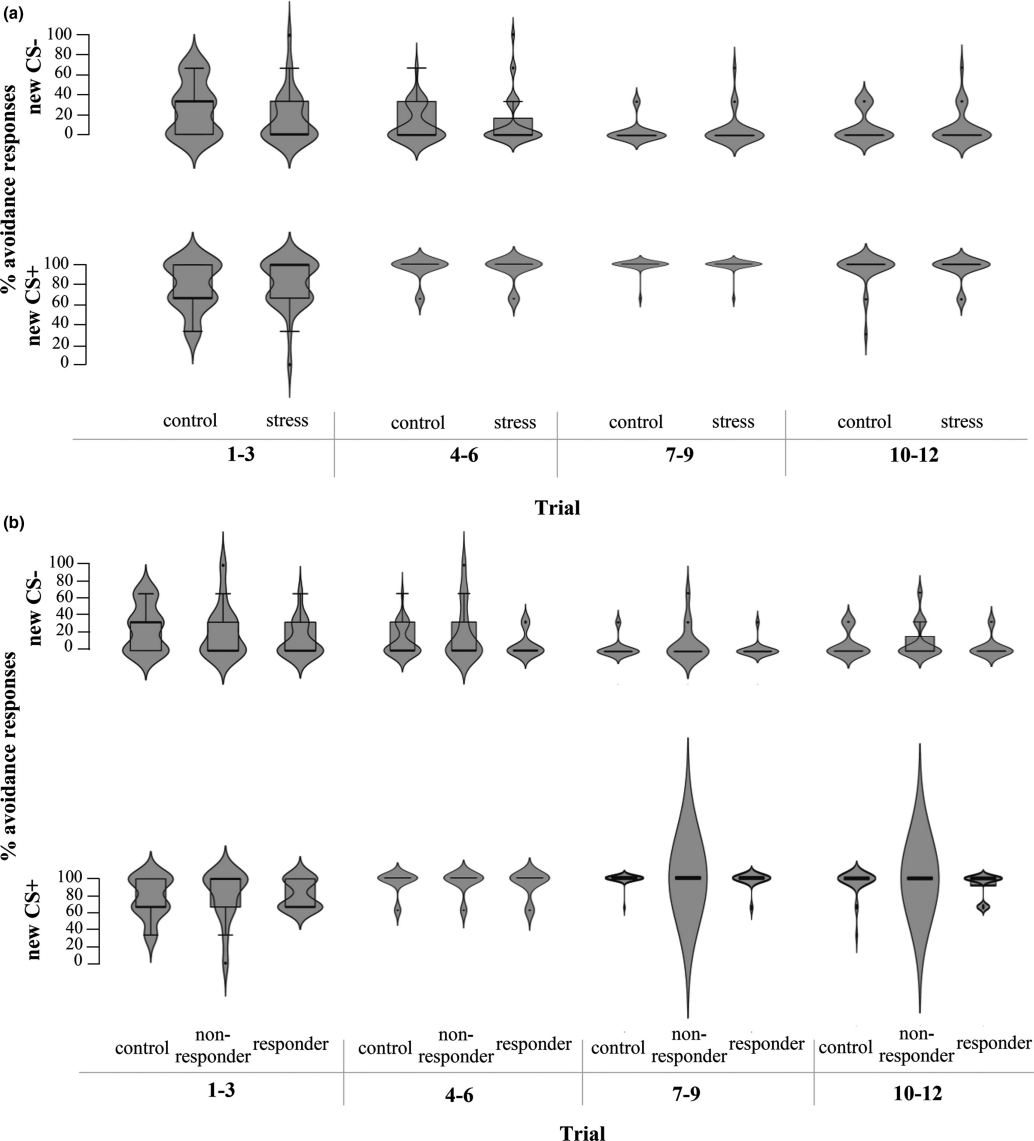
FIGUREPercentage of avoidance responses to the new CS‐ (red) and CS+ (green) (± SE) during trials 1–12 of the Reversal Test. There were no significant group differences. (a) Comparison stress and control group, (b) Comparison controls, cortisol non‐responders and responders

For avoidance responding, a GLM repeated measures ANOVA with Group (stress versus. no‐stress control) as between‐subjects variable and Stimulus (CS+ versus. CS‐) and Time (Reversal trials 1–3 versus. 4–6 versus. 7–9 versus. 10–12) as within‐subjects variables on percentages of avoidance revealed a significant Time*Stimulus interaction (*F*(2.07,122.01) = 23.50, *p* <.001, η*_p_* ^2^ = 0.29). Follow‐up analyses of Time per stimulus revealed a significant Time effect for both stimuli (*F*s > 12.40, *p*s < 0.001, η*_p_* ^2^ > 0.17). For the green dot (new CS+, previously CS‐), there was only a significant increase in avoidance responses from trials 1–3 to trials 4–6 (*p* <.001). For the red dot (new CS‐, previously CS+), the avoidance responses only decreased between trials 4–6 and trials 7–9 (*p* =.03). There were no differences between groups (all interactions and main effect: *F*s < 1.09, *p*s > 0.34; see Figure 5a). However, the analysis with ResponderType revealed a significant main effect (*F*(2,58) = 4.37, *p* =.02, η*_p_* ^2^ = 0.13). Follow‐up pairwise comparisons showed that cortisol responders pressed the avoidance button less often (i.e., *M* = 48.70, *SE* = 1.43) compared to cortisol non‐responders (*M* = 54.72, *SE* = 1.47, *p* =.01). Controls (*M* = 52.08, *SE* = 1.04) did not differ in the percentage of pressing the avoidance button from cortisol responders (*p* =.18), and cortisol non‐responders (*p* =.45; see Figure 5b).

### 8.5 Reversal index

A positive reversal index (RI) indicates more avoidance in response to the (new) CS+ during the Reversal Test relative to the final block of Avoidance Acquisition, whereas a negative index indicates more robust avoidance learning during the Avoidance Acquisition phase (Raio et al., 2017). For US‐expectancy RI, the one‐way ANOVAs revealed a significant difference between groups (*F*(1,59) = 5.14, *p* =.03), with stressed participants showing a positive RI, while controls participants had a negative RI. There was no significant difference between responder types (*F*(2,58) = 2.76, *p* =.07). For avoidance responding, two one‐way ANOVAs revealed no significant RI difference between the stress and the no‐stress control group (*F*(1,59) = 0.21, *p* =.65), nor between cortisol responders, non‐responders, and controls (*F*(2,58) = 0.97, *p* =.39).

Bivariate Pearson correlations yielded a significant group difference in correlations between the avoidance RI and alpha‐amylase measured immediately before the Reversal Test (t_+10_: *Z* = 2.912, *p* =.002), but not between the avoidance RI and alpha‐amylase before the stress induction and 20 min after the MAST (t_pre‐stress_: *Z* = −0.941, *p* =.17; t_+20_: *Z* = −0.99, *p* =.16). The stress group's avoidance RI was negatively correlated with alpha‐amylase levels measured immediately before the reversal test (t_+10_; (*r*(31) = −0.59, *p*_corrected_ = 0.01). However, in the control condition, this association was absent (*r*(30) = 0.11, *p*_corrected_ = 0.60). No other associations between the psychophysiological measurements (and change scores) and the reversal indices were significant in both groups (all *p*s_corrected_ > 0.14).

## 9 DISCUSSION

The aim of the current study was to investigate the effect of acute stress on habitual avoidance responding. The current results indicated that, following effective differential fear and avoidance learning, participants successfully updated the stimulus–outcome contingencies over the course of the Reversal Test by quickly learning to respond to the new CS+ (i.e., significant increase from the first three to the next three CS+ trials). Regarding reversal learning, participants learned to withhold a response after the first six new CS‐ trials of the Reversal Test. Moreover, results indicated that our stress manipulation was successful in eliciting subjective stress, more negative emotions and anxiety, higher cortisol responses, and increases in blood pressure levels. However, in contrast to our main hypothesis based upon the earlier findings of Raio and colleagues (2017) that stress would affect habitual avoidance responding, the current study did not demonstrate that acute stress leads to perseverance of avoidance responses. On the contrary, our results suggested more flexible updating of US expectancies after stress.

The current findings suggesting no difference between the stress and no‐stress control group in habitual avoidance responding during the Reversal Test are in line with the findings of Raio et al. (2017). In this study, stress did not seem to result in a failure to extinguish threat responses to a stimulus that no longer predicted danger, as evidenced by the absence of group differences in CS‐ responses during reversal. However, Raio and colleagues (2017) did find that participants in the stress condition had lower SCRs to the new CS+ during the reversal phase, indicating a failure to flexibly assign threat value to a stimulus that was previously safe. Taken together, this suggests that arousal might not specifically increase habitual responding, but instead leads to less flexibility in the updating of responses to changes in the environment or situation. However, when looking into the HPA‐axis stress responding by comparing cortisol responders and non‐responders, we found that participants displaying stress‐induced cortisol response pressed the avoidance button less often in comparison to cortisol non‐responders. Moreover, we also found that participants in the stress group rated the new contingencies more in line with the actual contingencies compared to the no‐stress control group. The finding that participants in the stress group seemed to be more alert and aware of the new contingencies might be explained by the fact that stress enhances attention and thereby biases cognition to central details and threat‐related information. The superior performance in the stress group also coincides with the observation that stress improved performance on simple tasks, like conditioning for negative stimuli (Luethi et al., 2009) that rely on basal ganglia circuits, the amygdala, and the hippocampus (Arnsten, 2009). These areas are part of the salience network that promotes vigilance, detection of threats, and stimulus–response behavior (Hermans et al., 2014; Seeley et al., 2007). The rapid increases of noradrenaline after stressor onset upregulate this salience network at the expense of the executive control network (Hermans et al., ,,^2011^, ^2014^; Schwabe, 2017). Thus, in our study, stressed or aroused participants might have performed more in accordance with the new contingencies, as they were more focused on new information regarding the CS+ and CS‐, which were related to the US.

The negative relationship between the reversal index and alpha‐amylase levels of stressed participants in the current study and the study by Raio and colleagues (2017) suggests the importance of noradrenergic activity in inflexible updating of stimulus–outcome associations. Taken together, these findings suggest that noradrenaline, and not cortisol, might be a driving force behind the inflexible updating of avoidance responses and coincides with previous research suggesting higher levels of noradrenaline in response to stress may impair prefrontal function and hence the flexible updating of avoidance responses to the new contingencies (Raio & Phelps, 2015). However, the correlation with alpha‐amylase should be interpreted with caution since no group and responder type differences were found in absolute alpha‐amylase levels. Alpha‐amylase levels were measured together with cortisol levels (t_pre‐stress_, t_+10_, t_+20_), even though noradrenaline and cortisol levels follow different patterns after exposure to a stressor (Joëls & Baram, 2009). Because alpha‐amylase is a measure of the fast, noradrenergic stress response, adding a measurement of alpha‐amylase levels during the MAST would have increased the sensitivity to detect a group difference in alpha‐amylase levels. The fact that we found no difference in reversal indices between the groups and responder types might seem to be in conflict with the findings by Raio et al. (2017). Yet their findings are based on SCRs, an outcome measure on a different response level of emotional arousal. Importantly, they are more closely linked to sympathetic nervous system activation and hence noradrenergic activity (Wickramasuriya & Faghih, 2020). SCRs, in contrast to US expectancy and avoidance responses, tap into different memory systems and do not always converge (e.g., Schultz et al., 2013). However, there are also studies showing that SCR conditioning only takes place in contingency aware participants and are therefore strongly related (e.g., Sevenster et al., 2014). For future studies, we recommend assessing SCRs in addition to the US expectancy and avoidance measures.

The lack of an effect of glucocorticoid responses in our study is not in line with the findings of instrumental learning studies (e.g., Schwabe & Wolf, 2009, 2010; Smeets et al., 2019). Smeets and colleagues (2019) found that cortisol responders made more errors to devalued outcomes in a slips‐of‐action test in comparison to cortisol non‐responders and controls. This suggests that habitual responding in instrumental learning tasks is driven by cortisol. In contrast, our results and those found by Raio et al. (2017) suggested that noradrenaline impairs the flexible updating of (avoidance) responses after stress. First, this discrepancy in results might be explained by the fact that the studies used other types of tasks. Whereas Schwabe and Wolf (2009, 2010) and Smeets and colleagues (2019) used appetitive instrumental learning paradigms and outcome devaluation, Raio et al.’s (2017) and our study designs involved threat learning and the reversal of contingencies. It is possible that different mechanisms are involved in reward versus threat learning and that stress has an effect on both these mechanisms. Moreover, methodological differences might at least partly explain the divergent findings. For example, the aforementioned instrumental learning studies used a task in which contingencies between actions and outcomes were more ambiguous (Schwabe & Wolf, 2009, 2010). An advantage of this partial reinforcement schedule is that it makes habits more resistant to extinction (Dickinson, 1985). In addition, it is assumed that besides overtraining (Tricomi et al., 2009), time pressure is one of the factors that favors habitual performance (De Houwer et al., 2018). In the current study, participants had 1000 ms to respond to the stimulus. In other paradigms used to investigate habitual responding response times were shorter or of equal duration, but for more complex tasks (e.g., De Wit et al., 2018; Gillan et al., 2014). Thus, it might be the case that the simplicity of our task in combination with the response time allowed participants to use the goal‐directed system, whether they showed a cortisol response or not. This is in line with the alternative dual process model by Moors et al. (2017), which states that goal‐directed processes are the primary determinant of behavior. Future studies could employ shorter response times (e.g., 500 ms) and make the task more difficult and ambiguous (e.g., more than two stimuli and a lower probability of the US) in order to increase reliance on the habitual system.

A few limitations of the current study are worth mentioning. First, we included female participants independent of hormonal contraceptives use. In future studies, we would recommend to either test women not using hormonal contraceptives during their luteal phase or to only include users of hormonal contraceptives, as studies have shown that hormonal alterations throughout the menstrual cycle are related to variability in cortisol responses after acute stress in women (e.g., Kudielka et al., 2009; Strahler et al., 2017). Note, however, that including such strict selection criteria would decrease the generalizability of the results to the general population. Second, avoidance responses in the Reversal Test phase reached a ceiling within seven to nine trials. This indicates that the task involving only two stimuli was rather easy. It is possible that the task might not have been sensitive enough in order to detect subtle changes induced by the stress manipulation. In future studies, we recommend increasing the difficulty of the task, as described in the previous paragraph. Third, the current study relied on a sample of healthy undergraduate students. Although the homogeneity of the sample is beneficial for studying the effect of stress on habitual avoidance responding, findings may not translate directly to clinical populations. Finally, it should be noted that due to the Covid‐19 pandemic we had to terminate recruitment and testing earlier than planned, leaving a few participants untested. However, given that our main findings were not even close to significance, we do not expect that adding the remaining seven participants would have led to different results.

In conclusion, the current study failed to demonstrate that acute stress leads to more habitual avoidance responding. On the contrary, results showed better stimulus–response awareness in a Reversal Test when under stress. Furthermore, results suggested that impairments in the flexible updating of avoidance responses are related to increased noradrenergic activity in stressed participants. Thus, it might be the case that not stress in general or the well‐studied cortisol response, but the noradrenergic response is accountable for shifting the balance toward inflexible responding. For this reason, we recommend to also include alpha‐amylase and skin conductance measurements in future studies on inflexible avoidance responding. Given that avoidance behaviors are one of the core symptoms of anxiety‐ and trauma‐related disorders that have a profound impact on the daily lives of patients (American Psychiatric Association, 2013), it is important to continue investigating factors that contribute to maladaptive avoidance behavior.

## CONFLICT OF INTEREST

The authors declare no conflict of interest.

## AUTHOR CONTRIBUTIONS

Anke Lemmens: Conceptualization, Methodology, Investigation, Formal Analysis, Visualization, Writing ‐ Original Draft; Conny Quaedflieg: Conceptualization, Methodology, Supervision, Formal Analysis, Writing ‐ Review & Editing. Pauline Dibbets: Conceptualization, Methodology, Supervision, Writing ‐ Review & Editing; Marleen Rijkeboer: Conceptualization, Methodology, Writing ‐ Review & Editing; Tom Smeets: Conceptualization, Methodology, Supervision, Writing ‐ Review & Editing.

### Peer Review

The peer review history for this article is available at https://publons.com/publon/10.1111/ejn.15155.

## Data Availability

All data, code, and stimuli are available upon request.
